# Analysis of feline and canine allergen components in patients sensitized to pets

**DOI:** 10.1186/s13223-016-0167-4

**Published:** 2016-11-30

**Authors:** Natalia Ukleja-Sokołowska, Ewa Gawrońska-Ukleja, Magdalena Żbikowska-Gotz, Ewa Socha, Kinga Lis, Łukasz Sokołowski, Andrzej Kuźmiński, Zbigniew Bartuzi

**Affiliations:** 1 Department and Clinic of Allergology, Clinical Immunology and Internal Diseases, Ludwik Rydygier Collegium Medicum in Bydgoszcz, NCU, ul. Ujejskiego 75, 85-168 Bydgoszcz, Poland; 2 Division of Ergonomics and Exercise Physiology, Department of Hygiene, Epidemiology and Ergonomics, Ludwik Rydygier Collegium Medicum in Bydgoszcz, NCU, ul. M. Curie Skłodowskiej 9, 85-094 Bydgoszcz, Poland

**Keywords:** Cat, Dog, IgE, Allergen components, Fel d 1, Lipocalin, Albumin

## Abstract

**Background:**

Component resolved allergen diagnosis allows for a precise evaluation of the sensitization profiles of patients sensitized to felines and canines. An accurate interpretation of these results allows better insight into the evolution of a given patients sensitizations, and allows for a more precise evaluation of their prognoses.

**Methods:**

70 patients (42 women and 28 men, aged 18–65, with the average of 35.5) with a positive feline or canine allergy diagnosis were included in the research group. 30 patients with a negative allergy diagnosis were included in the control group. The total IgE levels of all patients with allergies as well as their allergen-specific IgE to feline and canine allergens were measured. Specific IgE levels to canine (Can f 1, Can f 2, Can f 3, Can f 5) and feline (Fel d 1, Fel d 2, Fel d 4) allergen components were also measured with the use of the ImmunoCap method.

**Results:**

Monosensitization for only one canine or feline component was found in 30% of patients. As predicted, the main feline allergen was Fel d 1, which sensitized as many as 93.9% of patients sensitized to felines. Among 65 patients sensitized to at least one feline component, for 30 patients (46.2%) the only sensitizing feline component was Fel d 1. Only 19 patients in that group (63.3%) were not simultaneously sensitized to dogs and 11 (36.7%), the isolated sensitization to feline Fel d 1 notwithstanding, displayed concurrent sensitizations to one of the canine allergen components. Fel d 4 sensitized 49.2% of the research group.64.3% of patients sensitized to canine components had heightened levels of specific IgE to Can f 1. Monosensitization in that group occurred for 32.1% of the patients. Sensitization to Can f 5 was observed among 52.4% of the patients.

**Conclusions:**

Concurrent sensitizations to a few allergic components, not only cross-reactive but also originating in different protein families, are a significant problem for patients sensitized to animals.

## Background

Allergy to fur animals is a significant clinical problem for patients visiting allergologists. Allergies to felines and canines are most familiar to science, mainly because of their high occurrence frequency, tied to common exposure to allergens of these animals. In the USA alone, there are as many as 70 million dogs and 74.1 million cats living in households [1]. According to a 2012 study by TNS Poland about 48% of Poles own an animal. Among animal owners, in turn, 83% owns a dog, and 44% a feline [2].

The frequency of the occurrence of animal allergies in both Europe and the US has been undeniably rising for the last few decades [3]. According to a large 2009 study by Heinzerling et al. for 3034 patients from 17 centres in 14 European countries the average frequency of positive skin prick tests (SPT) with canine allergens was 27.2%, and 26.3% for feline allergens. The highest number of positive SPTs with these two types of allergens was noted in Odense in Denmark (56.0% for canines and 49.3% for felines), and the lowest in Vienna, Austria—16.1 and 16.8% respectively. In Poland in the Łódź centre positive SPTs were noted in 34.7% of cases and in 23.8% were positive to cat allergens [4].

An interesting study by Bjerg et al. from 2015 analyzed levels of specific IgE to cat, dog and horse allergens among 696 Swedish children aged 11–12, with the use of the ImmunoCap method. Elevated levels of specific IgE were found in 259 children (37.2%). Within that group, 51% showed symptoms of sensitization to all three allergens, 23% to two, and 25% to one of the allergens [5].

Patients often inquire about the course of development of their allergies, their prognoses, as well as possibilities of a co-occurrence of allergies to other animals.

Latest developments in component resolved diagnosis can prove immensely helpful in answering these questions.

Component resolved diagnosis (CRD) is most definitely a breakthrough in allergology. It allows to conduct a detailed analysis of the patient’s sensitization profile, and is to ultimately lead to a complex and individualized treatment in each case.

Unfortunately, this method also has a few drawbacks. Most importantly, its high price and low availability (only few centres make use of it), as well as the fact that at its current stage CRD only allows to mark a few animal allergic components making a negative result not identical to an allergy-excluding one [6], are all significant drawbacks of the method.

Not all known allergen components are available commercially. Among feline allergens Fel d 1, Fel d 2, Fel d 4 are available, as Can f 1, Can f 2, Can f 3, and Can f 5 are among canine allergen components. These can be used for a quantitive marking of IgE levels in blood serum using the highly sensitive, immune-fluorescent method, where antigens are tied in solid-phase (ImmunoCap). Another way of evaluating the levels of IgE antibodies against animal allergens is the semiquantitive microarray ImmunoCap ISAC assay, which allows to mark the aforementioned 7 components of felines and canines, along with the total of 112 components from 51 allergen sources [7].

Testing specific IgE levels for selected allergen components is used in academic work, but its application in practice continues to be limited. However, approaches to allergological diagnostics are undoubtedly developing in that direction. Allergies are now less conceived of in relation to the source of the allergen, and more in relation to a specific protein causing specific symptoms. This approach carries immense clinical implications, allowing to partially determine a natural development of the allergic disease, and in the future will influence the choice of the most effective treatment.

## Method and research material

Seventy patients (42 women and 28 men, aged 18–65, 35.5 on average) with a positive history of pet allergy were selected from patients of the Department and Clinic of Allergology, Clinical Immunology and Internal Diseases in Bydgoszcz. Patients treated for serious, chronic diseases and on medication that could influence the results of this study, were eliminated from the research group. Thirty patients (average age 37, aged 18–65) with negative allergy observation tests were included in the control group.

A detailed allergological interview was conducted for each patient, and a physical examination was conducted along with a skin prick test with extracts of common perennial allergens, including feline and canine allergens, using the Allergopharma set.

Patients both from the control group and the research group had their blood samples taken to assess the total levels of IgE and allergen-specific IgE to feline and canine allergens. Levels of specific IgE to available allergen components of canines (Can f 1, Can f 2, Can f 3, Can f 5) and felines (Fel d 1, Fel d 2, Fel d 4) were also determined.

All immunological determinations were performed with the use of the highly sensitive, immune-fluorescent ImmunoCap method (Thermo Fisher Scientific). The levels of IgE were evaluated as heightened when they exceeded 0.35 kU/l, in accordance with common practice in the field [5, 6].

Statistical analysis: Mann–Whitney and Kruskal–Wallis test, with Dunn test post hoc analysis, where applicable. Analyses were prepared using the R program, version 3.3.1.

The study was approved by the local Bioethical Committee and was assigned a classification number: KB158/2014.

## Results

In the research group feline allergen extract skin prick tests of 69 patients were positive, canine allergen skin prick tests tested positive for 34 patients. Heightened levels of total IgE (exceeding 100 kU/l) were found in 55 patients (78.6%). Heightened levels of specific IgE (in accordance with the commonly accepted threshold of 0.35 kU/l) against dog allergen extract were found in 56 patients, and in 66 patients against cat allergen extract. Levels of IgE specific for dogs and felines exceeding 0.7 kU/l were found in 46 and 63 patients respectively. Heightened levels (≥0.35 kU/l) of specific IgE to at least one of the allergen components of felines were found in 65 patients, and against at least 1 allergic component of canines in 42 patients. Out of the research group, 3 patients did not have heightened levels of specific IgE to any canine and feline components, and for 21 (30%) of patients a monosensitization to only 1 canine or feline component was noted.

Table 1 below presents the characteristics of the study population, including the frequency of sensitizations to animal allergen components in the research group.

**Table 1:** Characteristics of the study population, including the frequency of sensitization to animal allergen components in the research group

Features	Study group, n = 70	
Gender	Male-28	Female-42
Age (mean, min. and max. value)	35.5; 18–65	
	33.3; 19–65	37; 18–65
Concentration of total IgE (mean, min. and max. value) in kU/l	806.3; 7.44–5375	
	502.4; 7.44–5000	1008.9; 13–5375

Canine allergen components: number of patients with allergen-specific IgE levels exceeding 0.35 kU/l		
Can f 1 (lipocalin)	28 (40% of the study group)	
	7 (25% of male)	21 (50% of female)
Can f 2 (lipocalin)	10 (14.3% of the study group)	
	1 (3.6% of male)	9 (21.4% of female)
Can f 3 (albumin)	16 (22.9% of the study group)	
	6 (21.4% of male)	10 (23.8% of female)
Can f 5 (prostate gland - kallikrein)	22 (31.4% of the study group)	
	7 (25% of male)	16 (38.1% of female)

Feline allergen components: number of patients with allergen-specific IgE levels exceeding 0.35 kU/l		
Fel d 1 (secretoglobin, a uteroglobin-like protein)	61 (87.1% of the study group)	
	27 (96.4% of male)	35 (83.3% of female)
Fel d 2 (albumin)	16 (22.9% of the study group)	
	6 (21% of male)	10 (23.8% of female)
Fel d 4 (lipocalin)	32 (45.7% of the study group)	
	10 (35.7% of male)	22 (52.4% of female)

Table 2 presents the concentration of IgE, both total and specific, in female and male patients. The parameters, that are gender dependent are the concentration of IgE specific to Fel d 1 (p = 0.042) and Can f 1 (p = 0.031).

**Table 2:** Concentration of IgE in male and female (Mann–Whitney test, results are considered statistically significant when p < 0.05)

Feature	Gender	N	Mean value	SD	Median	Min	Max	p
Total IgE	Female	42	1008.92	1565.08	322.65	13	5375	p = 0.286
	Male	28	502.35	932.87	263	7.44	5000	
IgE specific to canine	Female	42	16.91	33.28	1.45	0	100	p = 0.302
	Male	28	5.79	18.73	1.2	0	100	
*rCan f 1*	*Female*	*42*	*14.55*	*29.84*	*0.34*	*0*	*100*	*p* *=* *0.031*
	*Male*	*28*	*3.6*	*15.94*	*0*	*0*	*84.6*	
rCan f 2	Female	42	8.39	23.81	0	0	100	p = 0.157
	Male	28	3.11	16.4	0	0	86.8	
nCan f 3	Female	42	11.5	28.08	0.02	0	100	p = 0.077
	Male	28	1.85	5.72	0	0	28.4	
rCan f 5	Female	42	8.87	24.5	0.11	0	100	p = 0.165
	Male	28	1.32	5.75	0.04	0	30.6	
IgE specific to feline	Female	42	15.11	24.1	3.58	0	100	p = 0.644
	Male	28	17.21	24.66	3.67	0.01	100	
*rFel d 1*	*Female*	*42*	*11.83*	*22.98*	*3.62*	*0*	*100*	*p* *=* *0.042*
	*Male*	*28*	*18.93*	*24.74*	*6.47*	*0*	*100*	
nFel d 2	Female	42	7.81	22.46	0	0	100	p = 0.486
	Male	28	4.98	18.98	0	0	98.5	
rFel d 4	Female	42	4.84	8.13	0.62	0	37.2	p = 0.089
	Male	28	3.46	8.96	0.03	0	42.1	

Statistically significant results are written in italics

We also evaluated the concentration of IgE depending on age of the subjects. The analysis was limited by the size of the population, therefor we analyzed 3 age groups—Group A, 22 patients, age <25 years old; Group B, 26 patients age 25–40; Group C, 22 patients >40. We found that the concentration of IgE specific to cat allergen extract was age dependent (p = 0.032), with higher values in patients >40 than in patients <25 years old. Concentration of total IgE, IgE specific to dog allergen extract and to all evaluated allergen components were not age dependent.

**Fig. 1:**
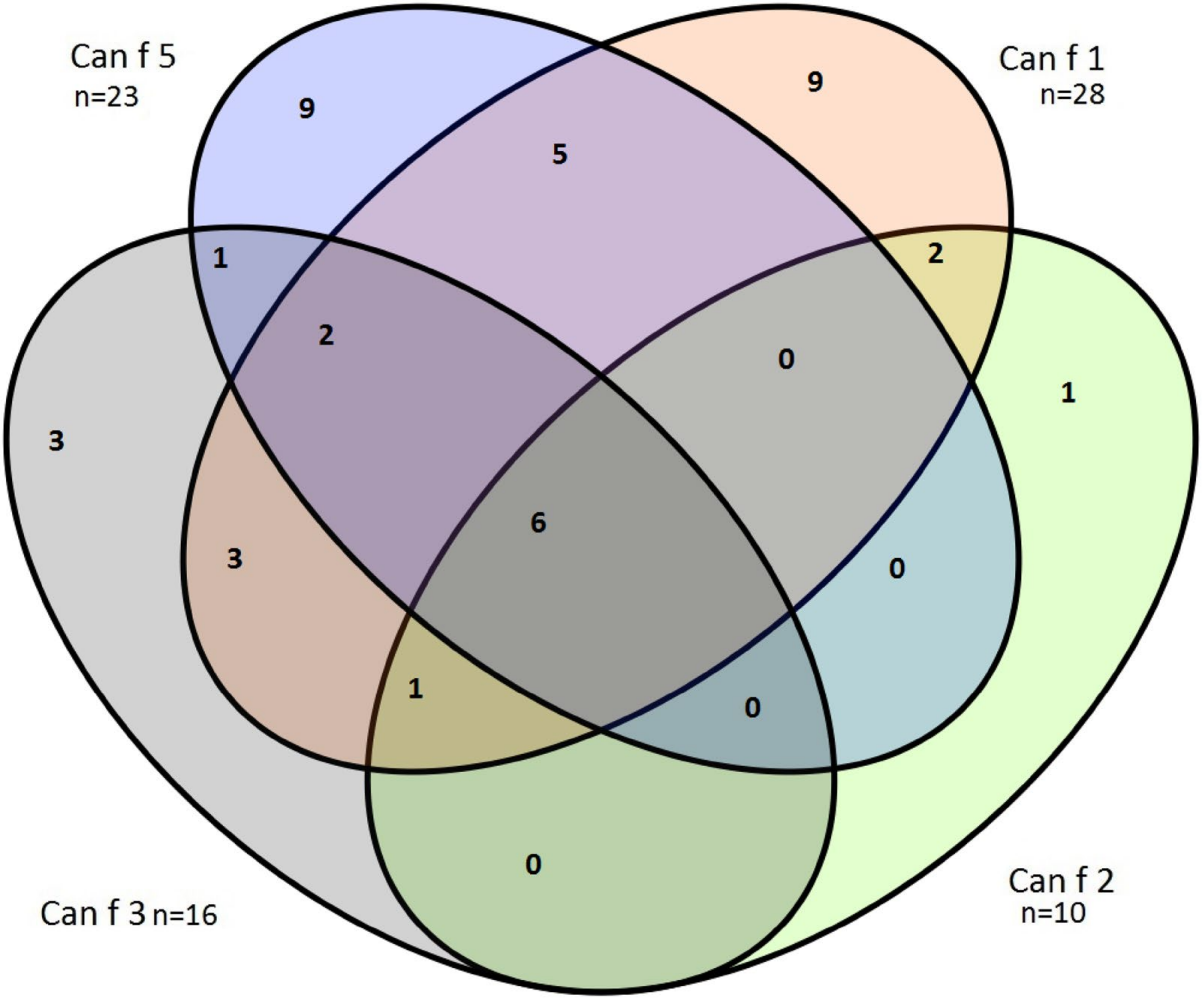
Co-occurrence of sensitizations to different canine allergen components (number of patients)

**Fig. 2:**
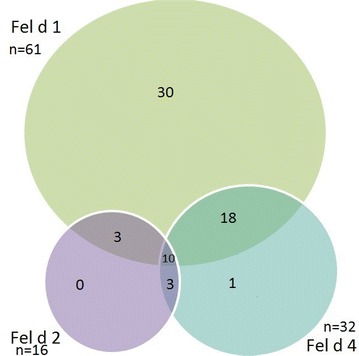
Co-occurrence of sensitizations to different feline allergen components (number of patients)

Both intra- and inter-specie co-occurrence of sensitizations to particular animal allergen components are here very important. Figure 1 illustrates the co-occurrence of sensitizations to different canine allergen components, and Fig. 2 to different feline allergen components. In both cases IgE levels exceeding 0.35 kU/ml were taken into consideration.

What draws attention here is the high frequency of allergies to both canine and feline allergen components in individual patients. It is worth noting that out of 32 patients sensitized to Fel d 4, as many as 28 (87.5%) had signs of sensitization to Fel d 1.

Fewest patients (16) had elevated levels of IgE to the Fel d 2 component. Within that group 13 (81.3%) had heightened IgE levels against Fel d 1, and 13 (81.3%) against Fel d 4.

Similar correlations can be found in the group of patients with sensitization to canines. The highest frequency of heightened IgE levels was noted for Can f 1 and Can f 5, main canine allergen components. Notably, heightened levels of specific IgE to Can f 2 were found in 10 patients, among which 9 had also sensitization to Can f 1, 7 patients in that group displayed sensitization also to Can f 3, and 6 out of these 10 patients were allergic to Can f 5.

Only 1 patient had an isolated sensitization to the Can f 2 allergen component. It is worth noting that as many as 12 (75%) out of 16 patients with heighted levels of specific IgE to Can f 3 (canine albumin) also sensitized to Can f 1.

A significant number of patients had co-occurring sensitizations to more canine components. As many as 6 had also sensitization to all 4 canine allergen components, sensitizations to 3 canine allergen components were found in 3 patients, sensitization to 2 canine components in 11 patients, and sensitization to one component in 22 patients out of 42 who had specific IgE to canine components.

Correlations between levels of particular canine and feline allergens in the research group were also studied. The findings show that there is a positive correlation between levels of canine allergen components (with the correlation ratio min. 0.7), but such a correlation was not noted for feline allergen components.

The correlation between the levels of each allergen component is presented in Table 3.

**Table 3:** The correlation between the levels of each allergen component in the research group

	rCan f 1	rCan f 2	nCan f 3	rCan f 5	rFel d 1	nFel d 2	rFel d 4
rCan f 1	1	0.90	0.77	0.71	−0.01	0.31	0.29
rCan f 2		1	0.77	0.82	−0.15	0.25	0.25
nCan f 3			1	0.75	−0.09	0.67	0.18
rCan f 5				1	−0.16	0.21	0.25
rFel d 1					1	0.11	0.44
nFel d 2						1	0.18
rFel d 4							1

An analysis of co-occurrence of sensitizations to each canine and feline allergen components is also highly important. Table 4 presents the number of patients sensitized to canine and feline cross-reacting allergens.

**Table 4:** The number of patients sensitized to canine and feline cross-reacting allergens

Type of protein	Feline allergen component	Number of patients with IgE levels ≥0.35 kU/l	Canine allergen component	Number of patients with IgE levels ≥0.35 kU/l	Co-occurrence of sensitizations to specific canine and feline components	Number of patients with IgE levels ≥0.35 kU/l for both components
*Lipocalin*	Fel d 4	32	Can f 1	27	Fel d 4 and Can f 1	20
*Lipocalin*	Fel d 4	32	Can f 2	10	Fel d 4 and Can f 2	9
*Albumin*	Fel d 2	16	Can f 3	16	Fel d 2 and Can f 3	15

**Graph 1:**
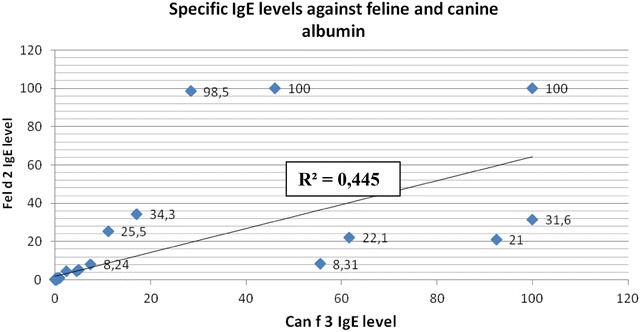
A comparison of specific IgE levels against feline and canine albumin

**Graph 2:**
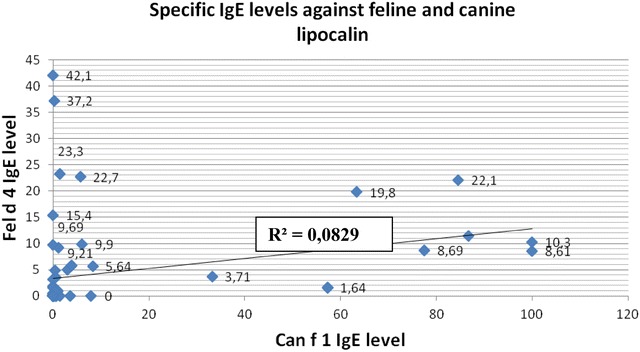
A comparison of specific IgE levels against feline and canine lipocalin

Interestingly, out of 10 patients with heightened levels of specific IgE to Can f 2, as many as 9 simultaneously had heightened levels of specific IgE to Fel d 4. Among the 16 patients sensitized to Fel d 2 and the 16 sensitized to Can f 3, as many as 15 (93.8%) had IgE antibodies reacting both with feline and canine albumin. No correlation between levels of IgE antibodies specific to albumin and lipocalin of canine and feline was noted, as illustrated on Graphs 1 and 2.

An analysis of the occurrence of an isolated sensitization to allergen components not commonly accepted as the reason behind cross-reactions also proves interesting. The main cat allergen, Fel d 1, a uteroglobin-like protein from the secretoglobin family, is an exception among mammal allergens, because in the majority of cases the main allergen is lipocalin. In our research group of 70 patients, 61 (87.1%) had heightened levels of specific IgE to Fel d 1. In the same group, 30 patients had elevated levels of IgE against Fel d 1, but with no specific antibodies against Fel d 2 and Fel d 4. Within that group 19 (63.3%) had an isolated sensitization to Fel d 1 among the examined animal allergen components. Curiously, 11 patients had heightened levels of IgE against Fel d 1, no specific antibodies against Fel d 2 (albumin) or Fel d 4 (lipocalin), but at the same time were sensitized to canine allergens.

Among canine allergen components Can f 5, a prostatic kallikrein, an arginine esterase, is of particular interest. In our research group 23 (32.9%) patients had elevated levels of specific IgE against Can f 5, 9 patients were sensitized only to Can f 5 out of all canine allergens, but simultaneously had symptoms of sensitization to feline allergen components; one patient had an isolated Can f 5 sensitization (only one among the canine and feline components under examination).

In the control group all participants had negative skin prick tests to animal allergens. No heightened levels of specific IgE to feline and canine allergens, or specific IgE to the canine and feline allergen components were found.

## Discussion

To our knowledge only few studies have addressed the clinical relevance of component resolved diagnosis in dog and cat allergy. The present study is focusing on the frequency of hypersensitivity to commercially available cat and dog allergen components in adult patients with symptoms of allergy to pets.

Fel d 1, thee main feline allergen, caused sensitizations in 93.9% of patients with sensitization to feline allergen components, from which group monosensitization was found in 30 (49.2%). A study by Bjerg et al. [5] found sensitization to the Fel d 1 component in 83.7% of its research group, which comprised of 208 children aged 11–12 sensitized to felines. In that group monosensitization was found in 67.8% of patients [5].

In the research group the second most frequent feline allergen was Fel d 4. Heightened levels of specific IgE for this feline lipocalin were found in 49.2% of patients. In the study by Bjerg et al. mentioned above, sensitization to Fel d 4 was found in 31.3% of the research group.

It is worth to emphasize, that in our population of adult patients the prevalence of polisensitization was higher than in the study population of Bjerg et al. It is consistent with findings of Asarnoj et al who found, that co sensitization to cat and dog allergen molecules becomes more common when patients are getting older. In their study children were examined during three time points (at 4, 8 and 16 years), and the level of IgE directed against allergen components were measured using microarray technology ImmunoCap ISAC. IgE reactivity to any of the 3 cat allergen molecules tested increased from 9.2% at 4 years up to 21.8% 16 years [8].

Results of The National Health and Nutrition Examination Survey (NHANES) 2005–2006 prove, that, in the USA population, production of IgE is dependent on sociodemographic factors, including gender and age. In NHANES 2005–2006 males seem more likely to have positive sIgE tests as well as elevated levels of sIgEs than females. What is more, inhalant allergen-specific IgEs peaked in childhood and early adulthood, declining in older age. According to authors it may reflect changes in the immune system that accompany aging [9].

In the present study it is worth to emphasize, that although there is a difference in mean value of total IgE in female and male, it is not statistically significant (Table 2). The parameters that are gender dependent are the concentration of IgE specific to Fel d 1 (p = 0.042), with higher values in male, and Can f 1 (p = 0.031), with higher values in female. In our study concentration of IgE specific to cat allergen extract was age dependent (p = 0.032), with higher values in patients >40 than in patients <25 years old. In case of evaluated allergen components the concentrations of IgE were not age dependent, although we feel that further research, on large population of adult patients, is necessary.

Among canine allergens, sensitization to the Can f 1 component was the most frequent—heightened levels of IgE were found in 64.3% of patients sensitized to canine allergen components. Within that group monosensitization was found in 9 patients (32.1%). Sensitization to Can f 5 was found in 52.4% of patients. In the study by Bjerg et al. [5] out of 218 patients (39%) 85 were found to be sensitized to Can f 1, and 102 (46.8%) to Can f 5. Sensitization to Can f 2 occurred as monosensitization extremely rarely, which was confirmed by our findings (1 patient in the research group). The difference in amount of patients sensitized to Can f 1 and Can f 5 in our population may be gathered with multiple factors, including age of the population, different pattern of sensitization or different methodology of the research [5].

Assessing levels of specific IgE to canine and feline allergen components can be helpful in making prognoses about patients who experience allergy reactions after contact with those animals. Some animal allergy components can cross-react both intra- and inter-species.

Information about a specific canine or feline protein which causes the sensitization carries important clinical implications. Relying on knowledge of the component source of the sensitization, theoretically, a potential risk of a cross-allergy, the potential a-typicality of the allergy, or even risks of infertility can be determined. In reality, however, things are, unfortunately, different. The above study points out the importance of allergy co-occurrence as a medical problem, not only among lipocalins or albumins, but also as a co-occurrence of an allergy to proteins belonging to different families. Undoubtedly this factor contributed to an obscuring of the correct clinical view of a case.

It seems that among patients sensitized to felines, with heightened levels of specific IgE to the main allergen Fel d 1, but who do not have specific IgE to other feline components, the risk of a cross-allergy to other fur animals is low, and is mainly related to allergies to rabbits. However, the above results indicate that among 65 patients sensitized to at least one feline component, for 30 (46.2%) it is the only sensitizing feline component, and within that group only 19 (63.3%) patients are not simultaneously sensitized to canines, with 11 (36.7%), despite an isolated feline Fel d 1 sensitization, having symptoms of a concurrent sensitization to some of the canine allergen components.

One of the explanations of this phenomenon may come from an interesting research by Reininger et al. It was the first report demonstrating the presence of an Fel d 1-like allergen in dog dander extracts. It was found that in 25% of Fel d 1-reactive cat-allergic patients (n = 36), more than 50% inhibition of IgE reactivity to dog allergens was achieved with recombinant Fel d 1 [10].

What is more, it seems that the isolated allergy to canine kallikrein is extremely rare. In our research group 42 patients had a sensitization to at least one canine component, 23 of them displayed symptoms of sensitization to Can f 5, and 9 out of these, in turn, out of all canine allergen components had an isolated sensitization only to Can f 5. Only one patient did not have a simultaneous sensitization to any of the feline allergen components.

An important observation seems, that there is a correlation between concentration of specific IgE to canine allergen components, but no such correlation is being observed in case of feline allergen components (Table 3). This phenomenon is not entirely understood. We know, that Can f 1 and Can f 2 have common epitopes and that most of the patients allergic to Can f 2 are co sensitized to Can f 1 [11, 12].

It remains partially unclear why in patients sensitized to fur animals the frequency of co-occurring allergies to a few components is high. Characteristically, this co-occurrence is related to components from different protein families. The high frequency of a co-occurring sensitization to various allergen components that we found in our research group confirms previous findings on this subject.

In a study by Liccardi et al. 900 people, 1/3 out of which had a sensitization to canines and felines and 1/3 were a non-atopic control group, the authors found that in the group of 300 people with sensitization to canines and felines there were significantly more people sensitized also to other fur animals in comparison to the other 300 patients sensitized to other allergens. Moreover, it was found that lack of immediate contact with a given animal (at home or through a hobby) does not exclude an allergy, indicating either a cross-reaction, or an ontogenetic propensity to an allergy to animal hair [13].

A 2015 study by Liccardi et al. conducted a similar analysis, relying on molecular diagnostics. They retrospectively analyzed results of an ISAC test from Allergy Unit, Fondazione Salvatore Maugeri, compiled in the course of 2 years. They divided patients into exactly the same groups as in the study described above (552 patients with a sensitization to canines or felines, 315 sensitized to inhalatory allergens different than canine or feline, and 189 patients with negative ISAC test results). They found that 42.9% of patients with sensitization to canines or felines had heightened levels of IgE to Equ c 1 (equine lipocalin) and Mus m 1 (mouse lipocalin). In other patient groups no heightened levels of specific IgE to Equ c 1 and Mus m 1 were found [14].

In our research group only 30% of the patients displayed monosensitization to 1 canine or feline component. Our results are also tangent with the latest study on the subject conducted by Uriarte et al. [12] which analyzed 159 patients with sensitization to animals. Only 5% of patients in that group were found to have monosensitization to a single allergen component; 86% of patients with sensitization to Fel d 4 were also sensitized to Fel d 1 [15]. In our study sensitization to Fel d 1 was found in 87.5% of patients sensitized to Fel d 4. Out of 10 patients found to have heightened levels of specific IgE to Can f 2, 9 also displayed heightened levels of specific IgE to Fel d 4.

There are a few drawbacks to this study. Although it was generally well conducted, with wide range of diagnostics, including skin prick tests and serum concentration of total IgE, specific IgE and IgE directed against currently available cat and dog allergen components (ImmunoCap), the results are based on only 70 adult patients. This study would benefit from extending the population, but it was limited by relatively high cost of allergen components in ImmunoCap, which were funded with an internal grant, number SLD-4/WL/2015 (Ludwik Rydygier Collegium Medicum in Bydgoszcz, NCU).

## Conclusion

Component resolved allergen diagnosis allows to improve diagnostics of sensitizations to animals. The primary drawbacks of this method are related to the high costs of marking IgE, as well as to the limited availability of particular allergen components. However, for particular patients an evaluation based on a detailed profile of sensitization to canines or felines and its correct interpretation may aid the process of determining the source of a-typical symptoms, and thus facilitate an accurate prognosis of the development of an animal fur allergy.

A highly relevant issue for most patients is the co-occurrence of sensitizations to a selection of allergen components, not only cross-reactive but also originating in different protein families. Monosensitivity to a specific allergen component occurs relatively rarely—our study noted it in 30% of tested patients.

## Abbreviations

SPT: skin prick test
CRD: component resolved diagnosis
